# Pathway to Remission in Severe Asthma: Clinical Effectiveness and Key Predictors of Success with Benralizumab Therapy: A Real-Life Study

**DOI:** 10.3390/biomedicines13040887

**Published:** 2025-04-06

**Authors:** Piotr Damiański, Adam Jerzy Białas, Marta Kołacińska-Flont, Anna Elgalal, Katarzyna Jarmakowska, Dorota Kierszniewska, Michał Panek, Grzegorz Kardas, Piotr Kuna, Maciej Kupczyk

**Affiliations:** 1 Clinical Department of Internal Medicine, Asthma and Allergy, Medical University of Lodz, 90-419 Lodz, Poland; marta.kolacinska-flont@umed.lodz.pl (M.K.-F.); aelgalal@poczta.onet.pl (A.E.); katarzyna.jarmakowska@umed.lodz.pl (K.J.); dorotaks@wp.pl (D.K.); michal.panek@umed.lodz.pl (M.P.); grzegorz.kardas@umed.lodz.pl (G.K.); piotr.kuna@umed.lodz.pl (P.K.); maciej.kupczyk@umed.lodz.pl (M.K.); 2 Department of Pneumology, Medical University of Lodz, 90-419 Lodz, Poland; adam.bialas@umed.lodz.pl; 3 Center for Allergy Research, Karolinska Institutet, SE-171 77 Stockholm, Sweden

**Keywords:** severe asthma, benralizumab, clinical remission, predictors, biologic therapy

## Abstract

**Introduction:** Recent data indicate that approximately 10–20% of patients with severe asthma (SA) receiving benralizumab (BENRA) do not achieve the desired outcomes. Emerging evidence suggests that clinical remission (CRem) is possible with biologics, warranting investigations into predictive factors. **Methods:** In this retrospective, single-center study, we analyzed 103 SA patients treated with BENRA for 12 months. CRem was defined as meeting four criteria: no exacerbations requiring oral corticosteroids (OCSs), discontinuation of chronic OCS therapy, improvement in FEV1 ≥100 mL, and an ACQ score < 1.5. Logistic regression identified predictors of remission. **Results:** After 12 months, 33% of patients achieved CRem, while 10% discontinued treatment due to lack of improvement. BENRA reduced the annual exacerbation rate from a median of 2 to 0 (*p* < 0.0001) and eliminated OCS use in 80% of patients. Lung function improved significantly, with a +13.5% predicted increase in FEV1 (*p* < 0.0001). Asthma control also improved, with ACQ scores decreasing from 3.2 to 1.5 (*p* < 0.0001) and mini-AQLQ scores increasing from 3.4 to 5.0 (*p* < 0.0001). Key predictors of remission included baseline eosinophil count ≥740 × 10^3^/μL (OR = 3.91, *p* = 0.02), SA duration (OR = 0.90, *p* = 0.02), baseline quality of life (OR = 2.18, *p* = 0.04), and pre-treatment FEV1 (OR = 1.07, *p* = 0.005). The logistic regression model for these parameters showed strong predictive accuracy (AUC = 0.855, 95% CI 0.78–0.93). Importantly, the SA phase, rather than total asthma duration, was the critical factor, with each additional year reducing the odds of remission by ~10%. **Conclusion:** Clinical remission is a realistic goal in severe asthma, and early initiation of biologic therapy is vital for improving remission rates and long-term outcomes.

## 1. Introduction

Severe asthma (SA), which affects approximately 5% of asthma patients—including both adults and children—is a complex and debilitating condition. It is characterized by frequent exacerbations and persistent symptoms despite treatment with high-dose inhaled corticosteroids (ICS) combined with a second controller and/or oral corticosteroids (OCSs), alongside appropriate management of contributing comorbidities and correction of factors such as poor adherence or improper inhaler technique. It also includes cases where asthma worsens when high-dose treatment is reduced. It significantly impairs patients’ quality of life, leading to physical limitations, psychological distress, and an increased risk of hospitalization, while also placing a substantial burden on healthcare systems. Generally, asthma management follows a stepwise approach, where treatment intensity is adjusted based on disease severity and response. First-line therapy includes ICS, with long-acting beta-agonists (LABAs) or leukotriene receptor antagonists (LTRAs) in moderate cases. The addition of long-acting muscarinic antagonists (LAMAs) is recommended in more severe cases of asthma. However, despite these therapies, many patients with SA remain symptomatic. They are forced to rely on chronic OCS use, exposing them to serious systemic complications such as osteoporosis, diabetes, hypertension, adrenal suppression, and increased infection risk [1,2].

Biologic therapies have revolutionized the management of SA by providing targeted interventions based on specific disease phenotypes and biomarkers. The Global Initiative for Asthma (GINA) guidelines recommend their use primarily to reduce OCS dependence and improve disease control in patients with uncontrolled severe type 2 inflammation [2]. Currently available treatments, including omalizumab, mepolizumab, benralizumab, reslizumab, and dupilumab, primarily modulate key effectors of the type 2 inflammatory cascade, such as immunoglobulin E (IgE) and cytokines: interleukin-5 (IL-5), IL-4, and IL-13. More recently, tezepelumab has emerged as a novel therapeutic option, offering earlier intervention in the inflammatory pathway by inhibiting thymic stromal lymphopoietin (TSLP). The growing array of biologics targeting different signaling pathways highlights the importance of precise phenotyping in optimizing treatment outcomes. The remarkable efficacy of these biologics has sparked increasing discussion on the feasibility of achieving clinical remission—a concept once considered unattainable [3,4,5].

Clinical remission, the primary objective in severe SA treatment, reflects a shift towards a more comprehensive approach that considers multiple complex indicators instead of single parameters. This applies to both predicting responses to therapy and evaluating treatment effectiveness. The concept of clinical remission on treatment is consistent with the long-term goal of asthma management promoted by GINA, emphasizing sustained disease control and improved patient quality of life. However, achieving CRem following biological treatment demonstrates considerable variability, ranging from 12% to 52.9%. This variability is likely due to the diverse criteria used to define remission, differences in study methodologies, and the heterogeneity of the patient populations studied [6]. Furthermore, many analyses rely on post hoc clinical trial data involving highly selected and controlled patient groups, failing to include a more diverse population or provide a complete picture of the disease. The same situation applies to predictors of remission, which vary depending on the studied population, biological drug, or even the country in which patients are treated [7]. Interestingly, a recent study on international variation in severe exacerbation rates among SA patients demonstrated that two patients with similar characteristics from different countries could have significantly different risks of future exacerbations [8]. This highlights the complexity of severe asthma management and the need for individualized treatment approaches. Understanding these variations can help optimize therapeutic strategies and improve patient outcomes globally.

Benralizumab (BENRA) specifically targets the IL-5 receptor, which plays a crucial role in eosinophil growth, activation, and survival. Clinical studies have consistently demonstrated BENRA’s effectiveness in reducing asthma exacerbations, decreasing OCS dependence, and improving quality of life and airflow parameters in targeted subgroups of patients with severe asthma (SA). However, despite these significant clinical benefits, approximately 10–20% of patients receiving BENRA fail to achieve a satisfactory therapeutic response. This underscores further research to identify predictive biomarkers and optimize personalized treatment strategies in SA management [9,10].

This study aimed to assess the real-world effectiveness of BENRA treatment in patients with severe eosinophilic asthma to determine the proportion of patients achieving clinical remission, clinical response, or non-response. Additionally, key predictive factors based on baseline characteristics were analyzed to identify determinants of clinical remission.

## 2. Materials and Methods

### 2.1. Study Design

This project was designed as an observational, retrospective, single-center study. It included patients who initiated biologic therapy between 17 February 2020 and 15 February 2023, beginning with a qualification visit for BENRA, followed by a one-year assessment to evaluate clinical remission rates, treatment effectiveness, and predictive factors of clinical remission. During the one-year treatment period, patients attended an average of seven scheduled visits at the clinic for therapy administration and routine clinical evaluation. Each visit included a short clinical assessment to monitor exacerbations and the use of OCSs, ensuring comprehensive disease control and treatment response evaluation.

Data from the qualification visit and patients’ hospital records were collected. For each participant, the following information was recorded: demographic details, asthma control status, exacerbations requiring short courses of systemic corticosteroids (or temporary increases in basal OCS dose) for at least three days, pharmacotherapy, time of asthma diagnosis, time of severe asthma diagnosis (according to GINA definition), mean daily OCS dose over the past 12 months, number of asthma hospitalizations, blood eosinophil count, skin prick tests (SPTs) or allergen-specific IgE (sIgE) and serum total IgE (tIgE)—if available—comorbidities, and associated treatments. Resting spirometry was conducted according to European Respiratory Society (ERS)/American Thoracic Society (ATS) standards at the qualification and one-year follow-up visits [11]. Quality of life was assessed using the Mini Asthma Quality of Life Questionnaire (mini-AQLQ), and asthma control was measured with the Asthma Control Questionnaire (ACQ) [12,13].

### 2.2. Inclusion and Exclusion Criteria

#### 2.2.1. Inclusion Criteria

Patients were eligible for inclusion in the analysis if they met all required qualification criteria for biological therapy and did not meet any exclusion criteria. To qualify for BENRA therapy, SA patients had to meet all of the following major criteria and at least two of the five minor criteria for severe, uncontrolled eosinophilic bronchial asthma, as defined by Poland’s National Health Fund (NHF) [14].

Major Criteria (all required):

- Blood eosinophil count (EOS) > 350 cells/µL within the past 12 months.
- At least two asthma exacerbations in the past year requiring OCSs for at least three days.
- High doses of inhaled ICS according to GINA guidelines [2], in combination with other controller medications.
- Non-smoker status.
- No history of hypereosinophilic syndrome or parasitic infestations.

Minor Criteria (at least two required):

- Uncontrolled asthma symptoms (ACQ score > 1.5).
- Hospitalization due to asthma exacerbation within the last 12 months.
- History of a life-threatening asthma attack.
- Persistent airway obstruction (FEV1 < 80% of predicted or daily PEF variability > 30%).
- Reduced quality of life due to asthma (mini-AQLQ score < 5.0).

#### 2.2.2. Exclusion Criteria

- The patient did not meet all major and at least two minor inclusion criteria.
- The patient had incomplete or missing baseline and/or follow-up data necessary for remission assessment, preventing reliable evaluation of treatment response.

### 2.3. Clinical Endpoints

The primary outcome was determining the proportion of patients achieving clinical remission (CRem), clinical response (CResp), or non-response (NResp), as well as identifying the key baseline factors influencing remission success in these patients. The CRem was defined based on four criteria: no need for OCSs, no exacerbations requiring OCSs or hospitalization, ACQ-6 score < 1.5, and an increase in pre-bronchodilator FEV1 ≥ 100 mL. CResp was defined in line with reimbursement regulations as an improvement by at least 0.5 points in both ACQ and mini-AQLQ scores, as well as a reduction in exacerbations and OCS use by more than 50%. The NResp group was defined by an absence of improvements in ACQ and mini-AQLQ scores and no reduction in exacerbations or systemic corticosteroid use greater than 50%. We further divided participants into groups, comparing the clinical characteristics of the CRem group with the non-remission (NRem) group—which included both the clinical response and non-response subgroups. The secondary outcomes included reductions in exacerbations requiring OCSs, maintenance OCS (mOCS) dose, and changes in pre-bronchodilator FEV1, mini-AQLQ, and ACQ-6 scores over 12 months of biologic treatment. Additionally, we compared the extent of improvement between patients who achieved clinical remission and those who did not.

### 2.4. Data Analysis

Statistical analysis of the data was conducted using R software v.4.4.2 for macOS. Data normality was verified with the Shapiro–Wilk test. Continuous variables were reported as means with standard deviations (SDs) or medians with interquartile ranges (IQRs) based on the data distribution. Comparisons between groups of patients CRem and NRem for biological therapy were performed using the unpaired Student’s *t*-test, Welch t-test, or Wilcoxon rank-sum test, depending on the normality of the data and homogeneity of variances. Paired data were analyzed using the Wilcoxon signed-rank test. For categorical variables, Pearson’s Chi-squared test or Fisher’s Exact Test was applied, as appropriate, based on expected frequencies. The cut-off point for eosinophil count was determined through receiver operating characteristic (ROC) curve analysis.

Logistic regression was employed to assess predictive factors of clinical remission. Forward and backward stepwise selection methods were used to refine the regression model. The model’s predictive performance was evaluated using ROC curve analysis, with the area under the ROC curve (AUROC) calculated to quantify its accuracy.

No corrections for multiple comparisons were applied, as all hypotheses and comparisons were pre-specified prior to data collection. Missing data were not substituted or estimated.

## 3. Results

### 3.1. Patient Demographic Data and Clinical Characteristics (Table 1)

In a cohort of 103 patients treated with BENRA, demographic, clinical, and comorbidity variables were compared between those in clinical remission (n = 34) and those not in remission (n = 69).

The mean age did not differ significantly between the CRem (60.6 ± 12.1 years) and the NRem groups (58.7 ± 12.6 years, *p* = 0.450). Similarly, the gender distribution was comparable, with proportions of 26.5% male in the CRem group and 37.7% male in the NRem group (*p* = 0.279). The median duration from asthma diagnosis to its progression to SA was 6 [1–16] years, with a tendency towards a longer duration in the CRem group (9 [3.5–19.5] years) compared to the NRem group (5 [1–14.5] years), approaching statistical significance (*p* = 0.07). Notably, the duration of SA was significantly shorter in patients in remission 5 [2–7] compared to those not in remission (9 [4–20] years, *p* = 0.003).

EOS was significantly higher in the remission group 755 [510–1015] than in the non-remission group (500 [420–810], *p* = 0.006), suggesting a potential association between eosinophil levels and clinical remission. There were no significant differences in body mass index (BMI) between the two groups (*p* = 0.47).

In terms of allergy markers, no statistically significant differences were found in sensitization rates to perennial allergens (23.5% in remission vs. 34.7% in non-remission, *p* = 0.35) or seasonal allergens (29.4% in remission vs. 27.5% in non-remission, *p* = 1.0).

Comorbidity analysis revealed a significantly higher prevalence of chronic rhinitis in the CRem group (88.2%) compared to the NRem group (60.9%, *p* = 0.008). However, no significant differences were observed in the presence of chronic rhinitis with polyps, the number of functional endoscopic sinus surgery (FESS)/polypectomy procedures, allergic rhinitis, cardiovascular disease, diabetes mellitus, gastroesophageal reflux disease (GERD), osteoporosis, Nonsteroidal anti-inflammatory drugs (NSAID) intolerance, or obesity between the two groups.

Regarding prior biological treatments, the use of omalizumab appeared to be higher in the non-remission group (23.1%) compared to the remission group (8.8%), although the difference was not statistically significant (*p* = 0.66). Similarly, the use of mepolizumab showed no significant difference, with rates of 2.9% in the remission group and 7.2% in the non-remission group (*p* = 0.105).

**Table 1:** Patient demographic data, clinical characteristics, and biomarkers.

Variables	Overall (n = 103)	Clinical Remission (n = 34)	No Clinical Remission (n = 69)	*p*-Value
Age, mean (SD)	59.3 ± 12.4	60.6 ± 12.1	58.7 ± 12.6	0.45
Male, n (%)	35 (33.9)	9 (26.5)	26 (37.7)	0.28
Total duration of asthma (non-SA +SA phase), median [IQR]	16 [10–32]	15 [11–23]	17 [10–34]	0.56
Duration from diagnosis of asthma to SA, median [IQR]	6 [1–16]	9 [3.5–19.5]	5 [1–14.5]	0.07
Duration of SA, median [IQR]	6 [2–13]	5 (2–7)	9 [4–20]	**0.003**
BMI, median [IQR]	27.5 [24–31]	28.5 [25.5–30.6]	27 [23.5–31.7]	0.47
EOS [cells/μL], median [IQR]	600 [430–885]	755 [510–1015]	500 [420–810]	**0.006**
Positive sIgE/SPT to perennial allergens, n (%)	32/103 (31)	8/34 (23.5)	24/69 (34.7)	0.35
Positive sIgE/SPT to seasonal allergens, n (%)	29/103 (28.1)	10/34 (29.4)	19/69 (27.5)	1
Number of polypectomy/FESS, mean (SD)	1 [0–2]	1 [1–2]	1 [0–2]	0.35
Reported comorbidities, n (%)				
Chronic rhinitis	72/103 (69.9)	30/34 (88.2)	42/69 (60.9)	**0.008**
Chronic rhinitis with polyps	37/103 (35.9)	14/34 (41.2)	23/69 (33.3)	0.66
Allergic rhinitis	33/102 (32.3)	9/35 (25.7)	24/68 (35.3)	0.53
Cardiovascular disease	47/92 (51)	15/32 (46.8)	31/60 (51.6)	0.83
Diabetes mellitus	7/103 (6.8)	2/32 (6.2)	5/69 (7.2)	1
GERD	8/95 (8.4)	1/33 (3.0)	7/62 (11.3)	0.27
Osteoporosis	6/97 (6.1)	0/34 (0)	6/63 (9.5)	0.17
Intolerance to NSAIDs	17/103 (16.5)	6/34 (17.6)	11/69 (15.9)	1
Obesity	32/100 (32.0)	8/32 (25.0)	24/68 (35.2)	0.42
Previously used biological treatment, n (%) Omalizumab Mepolizumab	19 (18.4) 6 (5.8)	3 (8.8) 1 (2.9)	16 (23.1) 5 (7.2)	0.66 0.11

Data are presented as the mean (SD), n (%), or median (25th percentile–75th percentile). Abbreviations: BMI, body mass index; GERD, gastroesophageal reflux disease; IQR, the interquartile range; FESS, functional endoscopic sinus surgery; NSAID, nonsteroidal anti-inflammatory drugs; SD, standard deviation; SPT, skin prick test, SA, severe asthma.

### 3.2. Baseline Asthma Control, Treatment, and Exacerbations (Table 2)

In terms of asthma treatment, the majority of patients received a combination of long-acting beta-agonists and inhaled glucocorticoids (LABA/ICS) (96%), with no significant difference between those in remission (97%) and those in the NRem group (95.6%, *p* = 1.00). The use of LABA/LAMA/ICS (4%), LAMA (33%), leukotriene receptor antagonists (LTRA) (52.4%), and theophylline (14.6%) did not differ significantly between the two groups (*p*-values: 1.00, 0.57, 0.89, and 0.374, respectively).

Regarding asthma exacerbations, patients in remission experienced fewer episodes requiring short courses of oral corticosteroids (OCSs) than those not in remission (*p* = 0.01). However, the hospitalization rates due to exacerbations over the previous year were similar between the two groups, with rates of 23.5% in the CRem group and 20.3% in the NRem group (*p* = 0.90).

Lung function assessments showed significantly better outcomes for patients in remission. The predicted FEV1(%) and FEV1 (L) were significantly higher in the remission group compared to the non-remission group (*p* = 0.0002 and *p* = 0.03 respectively). Additionally, asthma control and quality of life, as indicated by the ACQ and mAQLQ scores, were significantly better in patients in remission.

Chronic use of OCSs was significantly less common among patients in remission (8.8%) compared to those not in remission (29%, *p* = 0.02). Furthermore, the average daily OCS dose was notably lower in the remission group than in the non-remission group, a difference that also reached statistical significance (*p* = 0.007).

**Table 2:** Baseline asthma control, treatment, and exacerbations.

Variables	Overall (n = 103)	Clinical Remission (n = 34)	No Clinical Remission (n = 69)	*p*-Value
Asthma Treatment:				
LABA/ICS, n (%)	99 (96)	33 (97)	66 (95.6)	1.00
LABA/LAMA/ICS, n (%)	4 (3.8)	2 (5.9)	2 (2.9)	1.00
LAMA, n (%)	34 (33)	13 (38.2)	21 (30.4)	0.57
LTRA, n (%)	54 (52.4)	17 (50.0)	37 (53.6)	0.89
Theophyline, n (%)	15 (14.6)	3 (8.8)	12 (17.4)	0.37
Exacerbations requiring short courses of OCSs *, median [IQR]	2 [2–3]	2 [2–3]	3 [2–4]	**0.01**
Exacerbations requiring Hospitalizations in the preceding year, n (%)	22 (21.3)	8 (23.5)	14 (20.3)	0.90
FEV1(%) predicted, median [IQR]	62 [50.5–74]	70.5 [62.8–76]	58 [46–66]	**0.0002**
FEV1 (L) predicted, median [IQR]	1.65 [1.20–2.20]	1.80 [1.5–2.3]	1.60 [1.2–2.1]	**0.03**
ACQ score, median [IQR]	3.2 [2.8–3.8)	3.1 [2.8–3.4]	3.6 [2.8–4.1]	**0.03**
mAQLQ score, mean (SD)	3.31 (0.82)	3.58 (0.65)	3.17 (0.87)	**0.01**
OCS chronic use **, n (%)	23 (22.3)	3 (8.8)	20 (29)	**0.02**
OCS dose [mg/day] ***, median [IQR]	2.5 [1.2–5]	1.5 [1.0–4.12]	3.75 [1.5–7.25]	**0.007**

Data are presented as the mean (SD), n (%), or median (25th percentile–75th percentile). Abbreviations: ACQ, asthma control questionnaire; mini AQLQ, mini asthma quality of life questionnaire; FEV1, forced expiratory volume in 1 sec; ICS, inhaled corticosteroid; IQR, the interquartile range; LABA, long-acting beta-adrenoceptor agonist; LAMA, long-acting muscarinic antagonist, LTRA leukotriene receptor antagonists; OCSs, oral corticosteroid; M, mean; SD, standard deviation. * Intake of at least 3 days; ** Continuous intake of at least 6 months; *** The mean daily dose of oral corticosteroids, calculated in prednisone equivalents, during the year preceding qualification.

### 3.3. Clinical Remission (Figure 1a,b)

A total of 103 individuals who received BENRA were included in this analysis, with 10 (10%) discontinuing therapy after a year due to insufficient effectiveness. The majority of patients showed a clinical response, with 59 (57%) demonstrating significant improvement and 34 (33%) achieving clinical remission. Analysis of individual components of remission showed that 82% of patients did not have exacerbations requiring OCSs, 83% avoided maintenance OCSs for long-term disease control, and 74% achieved an FEV1 improvement of at least 100 mL. The most challenging component of CRem to achieve was an improvement in asthma control, as measured with the ACQ, with only 33% (n = 34) meeting this criterion. All outcomes were statistically significant compared to the 50% reference threshold, with *p* < 0.0001 for all components except the ACQ <1.5 (*p* = 0.0101).

**Figure 1:**
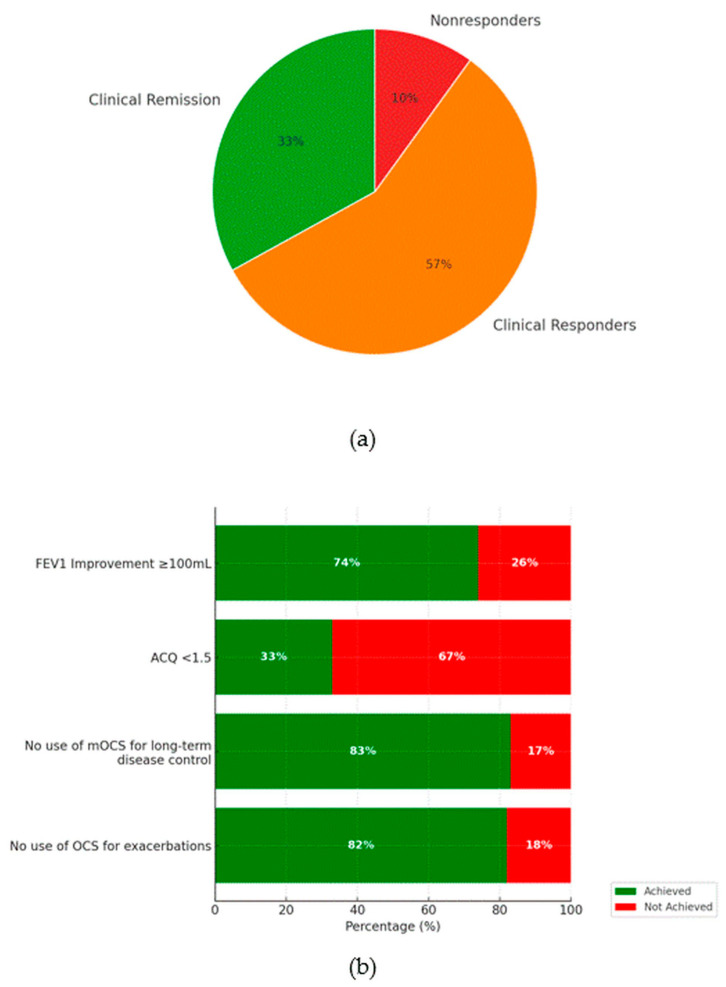
Panel (**a**) presents the proportion of patients meeting the criteria for clinical remission, clinical response, or classified as non-responders (%). Panel (**b**) illustrates the individual components of the disease remission criteria after one year of BENRA therapy (expressed as %).

### 3.4. Treatment Effectiveness

**Figure 2:**
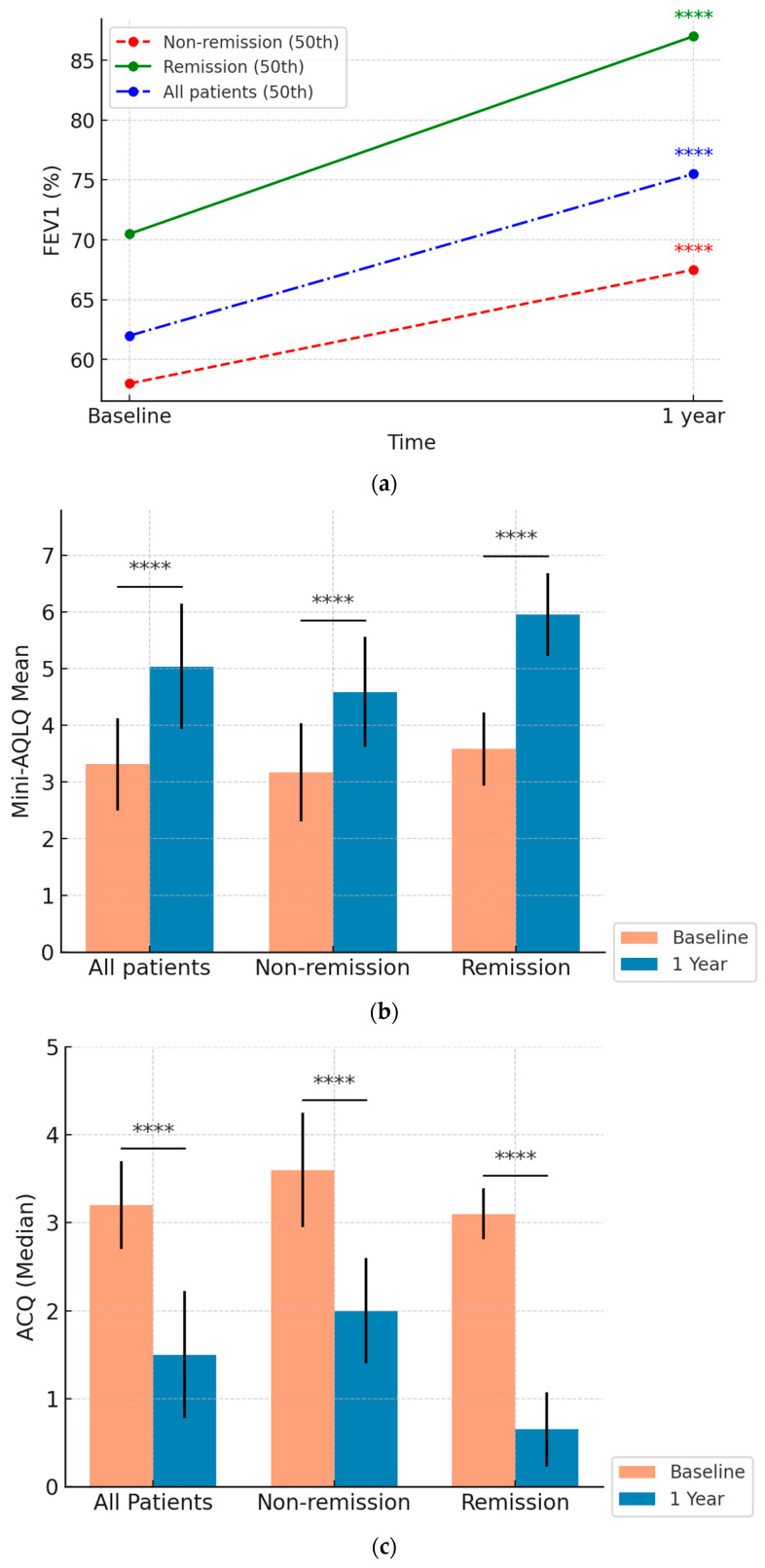
Illustrations of clinical improvement after one year of benralizumab treatment in each group (all patients, non-remission, and remission) (N = 103). The data highlight improvements in (**a**) median FEV1 (%) predicted, (**b**) Mini-AQLQ, and (**c**) ACQ-6 score. All within-group changes in FEV1, mini-AQLQ, and ACQ were statistically significant: Figure 2c—The bars represent medians; the error bars denote the interquartile range (25th–75th percentiles). **** = *p* < 0.0001.

**Figure 3:**
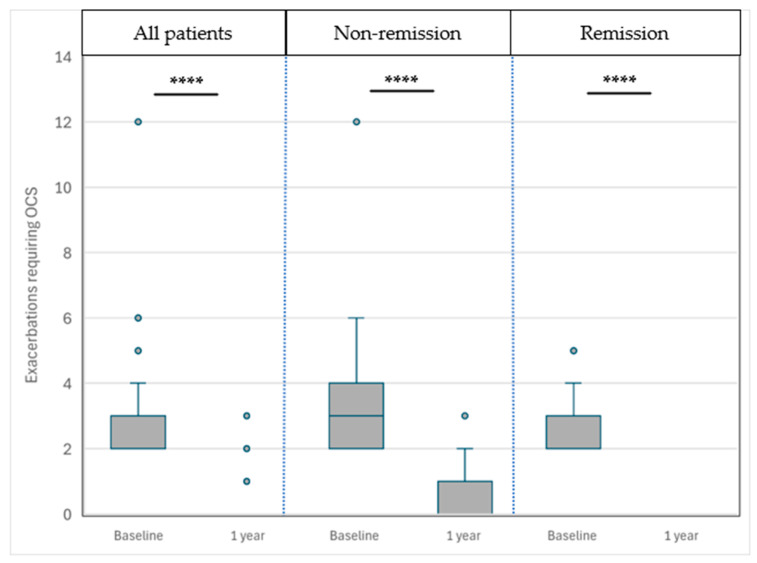
Comparison of exacerbations requiring OCSs before and after 1 year of treatment across the patient groups. The data are presented as medians with interquartile ranges (25th–75th percentiles) and whiskers representing the 5th and 95th percentiles, illustrating the distribution of exacerbations requiring OCSs before and after benralizumab treatment in the different patient groups, including all patients (non-remission + remission), non-remission, and remission groups. **** = *p* < 0.0001.

The analysis of 103 patients treated with BENRA over 12 months showed substantial improvements across multiple clinical metrics, with significant differences between the CRem and NRem groups (Figure 2 and Figure 3).

#### 3.4.1. FEV1 Improvement (Figure 2a)

Percentage and Absolute Change: FEV1 (%) significantly improved across all groups. The overall cohort improved from 62% [50.5–74%] to 75.5% [60–89%] in percentage predicted (*p* < 0.0001). Patients in the CRem group improved from 70.5% [62.8–76] to 87.0% [79.3–95], and those in the NRem group improved from 58.0% [46–66] to 67.5% [53.8–81.3] (both *p* < 0.0001).

The CRem group demonstrated a significantly greater increase in median FEV1 (%) compared to the NRem group: 12.5% [9–22.25%] vs. 9% [−1–18%], respectively (*p* = 0.04) (Supplementary Materials—Figure S1). Statistical analysis showed significant differences between the CRem and NRem groups at baseline in percentage-predicted FEV1 (*p* = 0.0002). These differences remained significant at 12 months (*p* < 0.00001).

#### 3.4.2. Quality of Life (Mini-AQLQ) (Figure 2b)

Quality of life, assessed using the Mini-AQLQ, improved significantly over 12 months. The overall mean mini-AQLQ score increased from 3.31 (0.82) points to 5.04 (1.11) points (*p* < 0.0001). Patients in the NRem group improved from 3.17 (0.87) to 4.58 (0.97), while those in the Crem group showed a greater increase from 3.58 (0.65) to 5.97 (0.73) (both *p* < 0.0001).

The CRem group experienced a more substantial increase 2.39 (0.87) vs. 1.41 (0.99) points (*p* < 0.0001) (Supplementary Materials—Figure S2). Baseline differences between the groups were statistically significant (*p* = 0.01), and post-treatment improvements were markedly greater in the CRem group compared to the NRem group (*p* < 0.00001).

#### 3.4.3. Asthma Control (ACQ) (Figure 2c)

Asthma control improved significantly across all groups. The overall ACQ score dropped from 3.2 to 1.5 over the 12 months (*p* < 0.0001). Patients in the non-remission group improved from 3.6 [2.8–4.1] to 2.0 [1.5–2.7], while those in the remission group improved more markedly from 3.1 [2.8–3.38] to 0.65 [0.3–1.15] (both *p* < 0.0001).

The CRem group experienced a more substantial decrease—2.6 [1.8–3.1] vs. 1.2 [0.6–2.2] points (*p* < 0.0001) (Supplementary Material—Figure S3). Differences between the CRem and NRem groups were statistically significant both at baseline (*p* = 0.03) and at the 12-month follow-up (*p* < 0.00001).

#### 3.4.4. OCS Dose Reduction

The use of oral corticosteroids (OCSs) was significantly reduced in both groups, especially in the CRem group. The overall cohort reduced their median OCS dose from 2.5 [1.19–5] mg/year to 0 [0–1] mg/year (*p* < 0.0001). The NRem group presented a higher reduction; however, the difference did not achieve statistical significance −2.5 [−5–−1.1] vs. −1.5 [−3.965–−1] (*p* = 0.16), which seems to be linked with a significant difference in baseline dose between the groups (*p* = 0.007), and there was a markedly greater reduction in the CRem group post-treatment (*p* < 0.00001).

#### 3.4.5. Frequency of Exacerbations Requiring OCSs (Figure 3)

The frequency of exacerbations requiring oral corticosteroids (OCSs) decreased substantially after one year of benralizumab treatment. In the overall cohort, the number of exacerbations was reduced from 2 [2–3] to 0 [0–0] per year. Patients in the NRem group improved from 3 [2–4] to 0 [0–1], while those in the CRem group improved from 2 [2–3] to 0 [0–0]. Both reductions were statistically significant (*p* < 0.0001).

Patients in the NRem group presented a significantly greater decrease in the number of such exacerbations −3 [−4–−2] vs. −2 [−3–−2] (*p* = 0.003).

### 3.5. Predictive Factors of Remission (Figure 4 and Figure 5)

Based on a significance threshold of *p* < 0.1, we observe the following statistically significant predictors for achieving clinical remission in asthma:

The presence of eosinophil count ≥740 × 10^3^/μL was associated with a 3.91-fold increase in the odds of remission compared to eosinophil counts <740 × 10^3^/μL (*p* = 0.02).

On the other hand, a longer duration of severe asthma was significantly associated with a reduction in the odds of remission (OR = 0.903, 95% CI: 0.831–0.981, *p* = 0.02). Specifically, each additional year of severe asthma reduced the odds of remission by approximately 9.7%. Over 10 years of SA translates into an approximately 63% reduction in the odds of remission, highlighting the substantial negative impact of prolonged severe asthma on the likelihood of achieving the outcome.

Higher pre-intervention Asthma Quality of Life Questionnaire scores were associated with a 2.18-fold increase in the odds of remission (OR = 2.18, 95% CI: 1.05–4.53, *p* = 0.04), suggesting that better baseline quality of life is predictive of more favorable outcomes. Similarly, a higher pre-FEV1 percentage of predicted was significantly associated with increased odds of remission, with each 1% increase in pre-FEV1 leading to a 7% increase in the odds (OR = 1.07, 95% CI: 1.02–1.12, *p* = 0.005). Finally, the model suggests that a higher average OCS dose in the last year before qualification—mg/day (pre-OCS use) may potentially reduce the odds of achieving the outcome by approximately 12.4% for each incremental unit (1 mg/day). The inclusion of pre-OCS improved the model’s discriminatory performance, as evidenced by an increase in the area under the ROC curve, indicating a better ability to distinguish between the outcome groups. Pre-OCS demonstrated a borderline significant association with the outcome (OR = 0.876, 95% CI: 0.756–1.01, *p* = 0.08). Despite its borderline significance, the model’s predictive performance improvement supported its inclusion.

The logistic regression model, using the factors of baseline EOS count, duration of severe asthma, pre-treatment quality of life, pre-treatment FEV1 (%), and OCS usage, demonstrated very good discriminative power with an AUC of 0.855 (95% CI 0.78–0.93). This result indicates that the model effectively and efficiently predicts the odds of remission.

**Figure 4:**
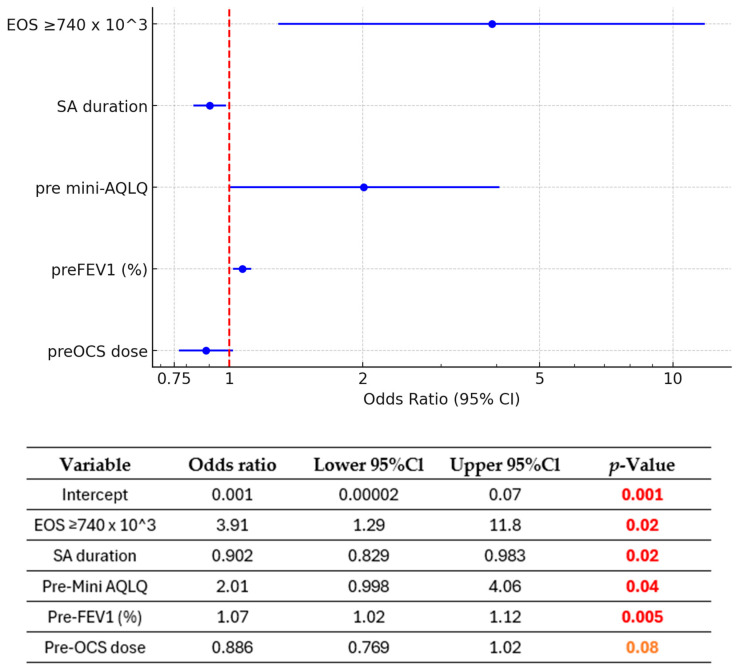
Association between pre-treatment characteristics and remission (multivariable analyses). EOS—blood eosinophil count; FEV1 (%)—pre-bronchodilator forced expiratory volume in one second, expressed as a percentage of predicted value; Mini AQLQ—Mini Asthma Quality of Life Questionnaire score; pre-OCS dose—mean daily oral corticosteroid dose calculated over the year preceding benralizumab initiation; SA duration—severe asthma duration (in years).

**Figure 5:**
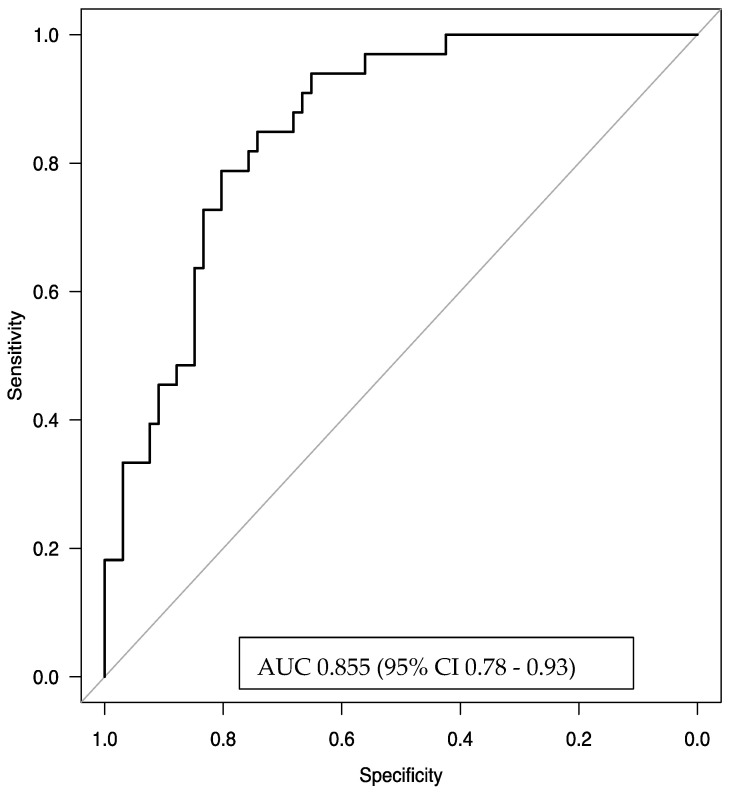
The area under the receiver operating characteristic (ROC) curve for the multivariate logistic regression model predicting clinical remission was 0.855 (95% confidence interval [CI]: 0.78–0.93), indicating strong discriminative ability. The explanatory variables included in the model were blood eosinophil count (EOS) ≥ 740 cells/µL, duration of severe asthma (in years), baseline Mini Asthma Quality of Life Questionnaire (Mini-AQLQ) score, pre-treatment forced expiratory volume in one second (pre-FEV1, expressed as a percentage of predicted value), and the average daily dose of oral corticosteroids (pre-OCS) a year before benralizumab initiation.

## 4. Discussion

Our analysis provides real-world insights into the effectiveness and key predictors of clinical remission after one year of BENRA treatment in a Polish cohort of SA patients. BENRA treatment led to clinical improvements in the majority of patients, with over one-third achieving clinical remission based on the five-point criteria proposed by Menzies-Gow et al. [15]. Most patients experienced enhanced lung function, fewer exacerbations requiring OCSs, reduced OCS dependence, and improved asthma control and quality of life. Clinical remission was predicted by a shorter duration of severe asthma, better baseline lung function, higher EOS (≥740 × 10³/µL), lower mean daily OCS doses, and better quality of life prior to therapy initiation. Patients with less severe disease status at baseline showed a more substantial treatment response. Conversely, around 10% of patients demonstrated insufficient clinical improvement, leading to their disqualification from continued anti-IL-5R therapy according to reimbursement criteria.

In our study group, 33% of patients with severe asthma achieved clinical remission, placing our results within the mid-range reported in the literature, which varies from 18% to 55% [6]. However, our outcomes were slightly better than those of studies using the same remission criteria. Specifically, the remission rates in the SIROCCO and CALIMA trials were 23.9%, while the ZONDA trial reported a rate of 32.5% (after six months of treatment) [15]. The UK Severe Asthma Registry (UKSAR) showed a remission rate of 18.3% across all biological therapies [16]. The ACQ < 1.5 was the most difficult criterion for remission, achieved by only 33% of our patients. In contrast, 50.3% and 67.5% of patients in the SIROCCO/CALIMA and ZONDA trials, respectively, met this criterion. This discrepancy likely arises from the poorer baseline scores in our group (ACQ = 3.4) compared to the scores of 2.87 and 2.7 in the SIROCCO and CALIMA trials, respectively [9,10,15,17]. Additionally, real-life studies also reported better baseline scores, such as 3.2 in UKSAR [16] and 2.52 in the Danish Severe Asthma Register (DSAR) [18]. These findings suggest that our patients had a higher baseline symptom burden, likely due to the stricter reimbursement eligibility criteria.

The treatment effectiveness data closely align with clinical trial findings, though some variation exists in the degree of improvement. Notably, BENRA led to a median increase in FEV1 of 13.5%, allowed for independence from OCSs, and reduced exacerbations requiring OCSs in over 80% of patients. These results surpass those reported in the SIROCCO, CALIMA, and ZONDA studies [9,10,17]. The key differences in outcomes result from our careful medication selection, which identified patients with significantly higher eosinophil levels (median 600/μL) compared to those seen in the SIROCCO and CALIMA studies (median 360–400/μL) [9,17]. This selection was based on precise phenotypic diagnosis conducted by specialists at one of Poland’s leading qualification centers. Frequent follow-ups under the National Health Fund drug reimbursement program (6–7 annual visits) further facilitated close monitoring and therapy adherence. This aligns with findings from specialized asthma centers elsewhere, where regular follow-ups and careful phenotypic assessment notably improved clinical outcomes, even independently of biologic therapies [19].

The predictive model developed in this study, incorporating eosinophil count, SA duration, Mini-AQLQ score, pre-treatment FEV1, and pre-treatment average OCS dose, provides a clinically relevant tool for assessing remission likelihood. This can enhance shared decision-making and align patient expectations with realistic outcomes.

In our study, FEV1 (%) emerged as an important predictor of clinical remission in SA, in line with the existing literature [16,18,20]. Although data regarding long-term changes in spirometric parameters in SA remain limited, the available literature reports a consistent annual decline in post-bronchodilator FEV1 of approximately −25.7 to −31.5 mL, with greater reductions observed in patients experiencing frequent exacerbations [21,22]. Importantly, this decline also occurs in younger adults, who typically maintain stable lung function (ages 25–40 years) [23]. Our results indicate that benralizumab treatment effectively reverses this trend, significantly improving FEV1 in both CRem and NRem patients, with the greatest benefits seen among those achieving remission. This improvement is likely due to BENRA’s positive indirect effect on airway remodeling, which reduces chronic inflammation and structural changes such as smooth muscle hypertrophy [24]. These findings raise the question of whether eligibility for biological treatment should incorporate the criterion of progressive lung function decline rather than relying exclusively on a fixed threshold (e.g., FEV1 < 80%).

The duration of severe asthma also proved to be a crucial predictor of remission. Interestingly, most studies fail to differentiate between non-severe and severe phases of asthma, leading to variability in findings. For example, the International Severe Asthma Registry (ISAR) and UKSAR reported that each additional 10 years of asthma before starting biologics decreased the odds of remission by 15% and 14%, respectively [16,25]. However, other studies do not support this trend [26]. In our analysis, the overall duration of asthma was not significantly associated with CRem. Instead, the time spent in the severe phase emerged as the decisive factor—each additional year reduced the odds of remission by approximately 10%. These findings highlight the need for prompt recognition and early therapeutic intervention in SA to improve remission rates and long-term outcomes.

Our multivariate analysis demonstrated that CRem was associated with a lower daily OCS dose in the year before qualification and higher EOS, both of which are well-documented in the literature [27]. Although introduced in 1999, the Mini-AQLQ remains a standard instrument for patient characterization and assessment of treatment effects in asthma [12,28]. In our study, quality of life measured using this tool emerged as a significant predictor, with each 1-point increase doubling the odds of clinical remission. Similar findings have been reported in Spanish cohorts [20], further emphasizing the relevance of patient-reported outcomes in guiding therapeutic decisions.

Regarding comorbidities, we found no statistically significant differences between the CRem and NRem groups, which may be attributed to the relatively small sample size of the study, potentially limiting the statistical power to detect such differences. Although obesity is commonly reported to decrease remission rates, our study did not find a statistically significant association despite a higher prevalence of obesity in the NRem group (35% vs. 25%, *p* = 0.42) [19,20,29].

The stricter reimbursement criteria in Poland likely contributed to the more severe baseline characteristics of our cohort compared to other registries, such as the DSAR and the UKSAR [16,18,29]. For example, in the DSAR, 25% of patients had ACQ scores below 1.5, whereas such patients would not qualify for biologics in Poland [18]. A similar situation occurred in a Spanish real-world study, where over 11% of SA patients were active smokers, a disqualifying factor for biological therapy in our country [20]. Additionally, differences in treatment access, such as the late reimbursement of fixed triple therapy in Poland (May 2022), may have influenced outcomes. These variations emphasize the need for country-specific analyses to optimize treatment strategies across different healthcare systems.

This study reflects the real-world experience of Barlicki University Hospital, a leading center for biological treatment under the Polish NHF program. With a large patient base, the hospital provides an ideal setting for evaluating the use and outcomes of benralizumab therapy. However, the study has some limitations, including its retrospective, single-center design and relatively small sample size, which limits the generalizability of the results. The COVID-19 pandemic further restricted the analysis by limiting access to spirometry and follow-up visits. Additionally, among patients’ phenoendotypic characteristics, only eosinophilic or allergic status was considered. Moreover, the follow-up period could have been longer to provide a more comprehensive assessment of treatment durability and long-term remission rates. Despite these challenges, our study covers approximately 10% of all SA patients treated with benralizumab in Poland by the end of the data collection period, making it the largest published database on the effectiveness of this therapy in the country [30]. Importantly, the reliability of the dataset is reinforced by its origin in the national drug program, ensuring standardized eligibility criteria, treatment protocols, and systematic data collection.

## 5. Conclusions

Clinical remission is an attainable goal in some SA patients and should be a standard treatment outcome. This real-world study confirms the effectiveness of BENRA in improving lung function, reducing OCS use, and enhancing both quality of life and disease control, particularly among patients in the CRem group, who presented with less severe disease status at baseline. Timely biologic therapy maximizes remission rates and improves long-term outcomes.

## Data Availability

Data will be shared upon reasonable request.

## Supplementary Materials

The following supporting information can be downloaded at: https://www.mdpi.com/article/10.3390/biomedicines13040887/s1, Figure S1: Median change in FEV₁ (%) after one year of benralizumab treatment in patients with and without clinical remission; Figure S2: Change in mean quality of life (Mini-AQLQ score) after one year of benralizumab treatment in patients with and without clinical remission. Figure S3: Change in asthma control questionnaire (ACQ) after one year of benralizumab treatment in patients with and without clinical remission.

## Abbreviations

The following abbreviations are used in this manuscript: ACQ—Asthma Control Questionnaire, ATS—American Thoracic Society, AQLQ—Asthma Quality of Life Questionnaire, BENRA—Benralizumab, BMI—Body Mass Index, CRem—Clinical Remission, EOS—Eosinophil Count, ERS—European Respiratory Society, ExOCS pre—Exacerbations Requiring Short Courses of Oral Corticosteroids Before Qualification, and ExOCS post—Exacerbations Requiring Short Courses of Oral Corticosteroids During 1 Year of Benralizumab Treatment. Other abbreviations include the following: FEV1—Forced Expiratory Volume in 1 Second, FESS—Functional Endoscopic Sinus Surgery, GERD—Gastroesophageal Reflux Disease, GINA—Global Initiative for Asthma, ICS—Inhaled Corticosteroids, LABA—Long-Acting Beta-Adrenoceptor Agonist, LAMA—Long-Acting Muscarinic Antagonist, LTRA—Leukotriene Receptor Antagonists, mAQLQ—Mini Asthma Quality of Life Questionnaire, NRem—Non-Remission, NSAID—Nonsteroidal Anti-Inflammatory Drugs, OCS—Oral Corticosteroids, PEF—Peak Expiratory Flow, SA—Severe Asthma, sIgE—Allergen-Specific Immunoglobulin E, SPT—Skin Prick Test, tIgE—Total Immunoglobulin E, ROC—Receiver Operating Characteristic, AUROC—Area Under the Receiver Operating Characteristic Curve, IQR—Interquartile Range, SD—Standard Deviation, CI—Confidence Interval, OR—Odds Ratio, AUC—Area Under the Curve, and NHF—National Health Fund.
