# Correction: Damiański et al. Pathway to Remission in Severe Asthma: Clinical Effectiveness and Key Predictors of Success with Benralizumab Therapy: A Real-Life Study. *Biomedicines* 2025, *13*, 887

**DOI:** 10.3390/biomedicines13102549

**Published:** 2025-10-20

**Authors:** Piotr Damiański, Adam Jerzy Białas, Marta Kołacińska-Flont, Anna Elgalal, Katarzyna Jarmakowska, Dorota Kierszniewska, Michał Panek, Grzegorz Kardas, Piotr Kuna, Maciej Kupczyk

**Affiliations:** 1 Clinical Department of Internal Medicine, Asthma and Allergy, Medical University of Lodz, 90-419 Lodz, Poland; marta.kolacinska-flont@umed.lodz.pl (M.K.-F.);; 2 Department of Pneumology, Medical University of Lodz, 90-419 Lodz, Poland; 3 Center for Allergy Research, Karolinska Institutet, SE-171 77 Stockholm, Sweden

## Error in Figure

In the original publication [1], there was a mistake in Figure 1b as published. In the section of the figure corresponding to ACQ < 1.5, the values are currently presented incorrectly. According to the text above the figure, the correct values should be “achieved: 33%”, “not achieved: 67%”.

The corrected Figure 1b appears below:

**Figure 1:**
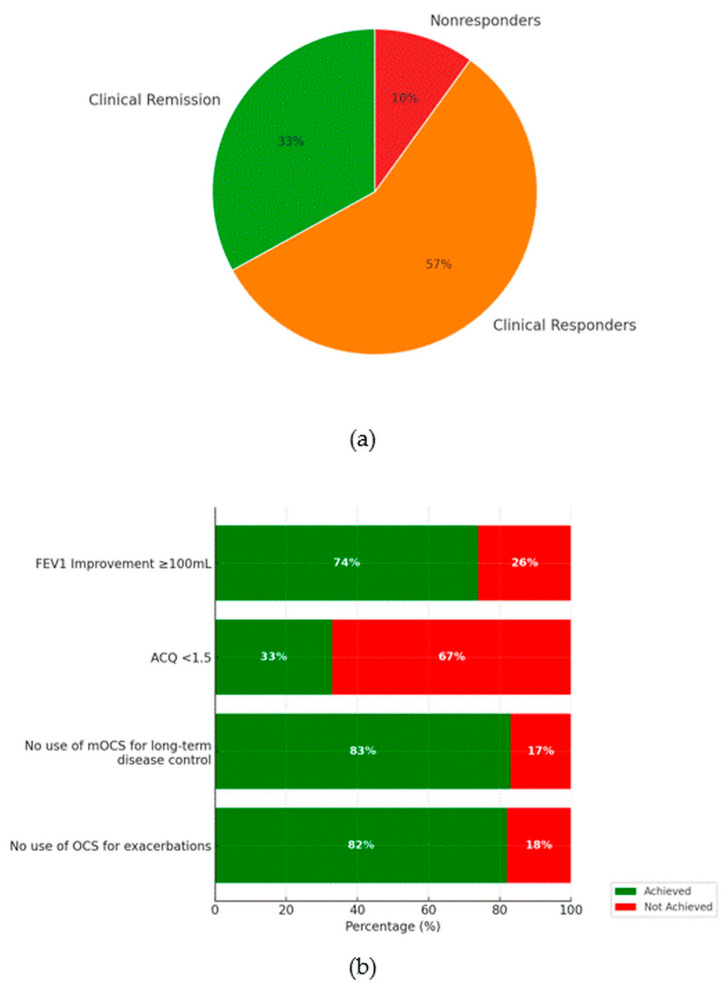
Panel (**a**) presents the proportion of patients meeting the criteria for clinical remission, clinical response, or classified as non-responders (%). Panel (**b**) illustrates the individual components of the disease remission criteria after one year of BENRA therapy (expressed as %).

The authors state that the scientific conclusions are unaffected. This correction was approved by the Academic Editor. The original publication has also been updated.
