# Supplementary material for: Pathway to Remission in Severe Asthma: Clinical Effectiveness and Key Predictors of Success with Benralizumab Therapy: A Real-Life Study
Source: Biomedicines. 2025 Apr 6;13(4):887. doi: 10.3390/biomedicines13040887 (PMC12024904; doi:10.3390/biomedicines13040887)
Supplement: Supplementary file 1 [file biomedicines-13-00887-s001.zip › biomedicines-3519382-supplementary.pdf]

Supplementary figures

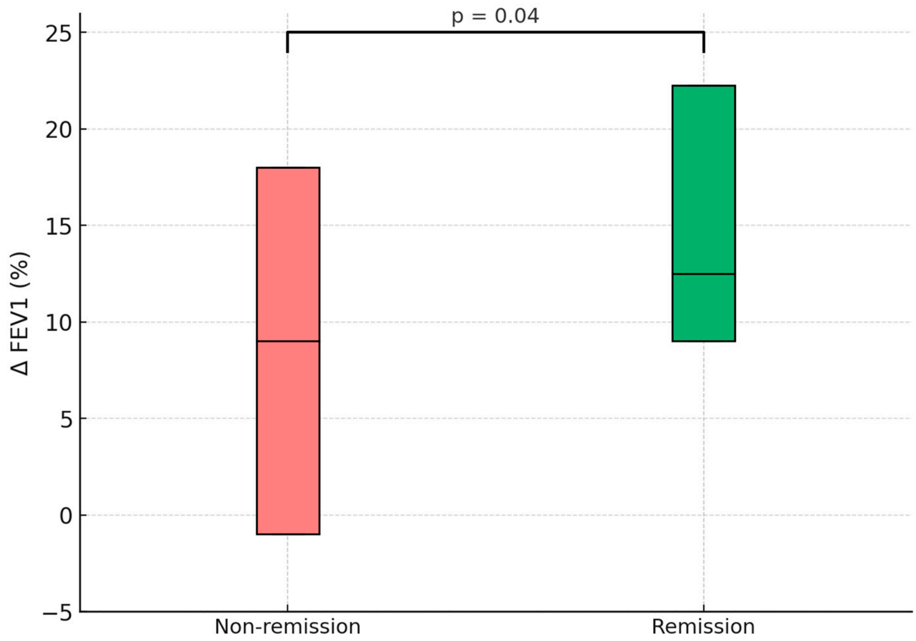

Supplementary Figure S1: Median change in FEV_1 (%) after one year of benralizumab treatment in patients with and without clinical remission. Boxplots represent median values and interquartile ranges (IQR), with the lower and upper bounds of each box corresponding to the 25th and 75th percentiles, respectively

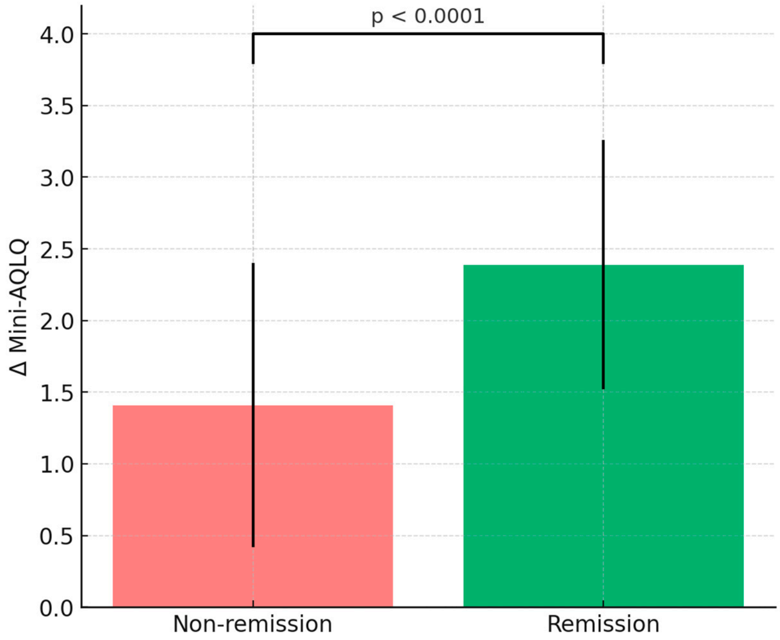

Supplementary Figure S2: Change in mean quality of life (Mini-AQLQ score) after one year of benralizumab treatment in patients with and without clinical remission.

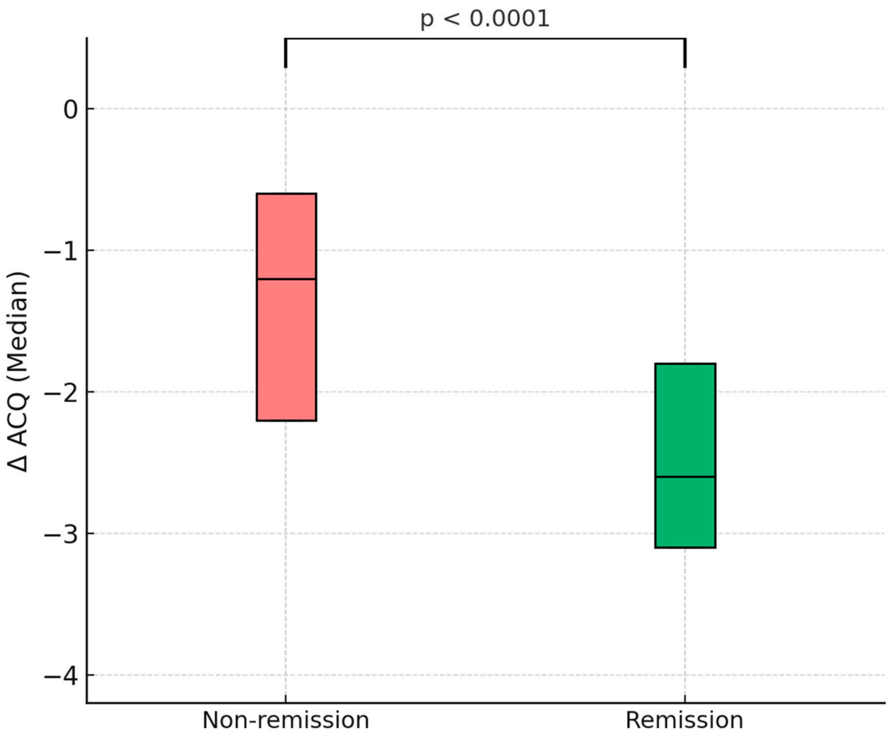

Supplementary Figure S3: Change in asthma control questionnaire (ACQ) after one year of benralizumab treatment in patients with and without clinical remission. Boxplots represent median values and interquartile ranges (IQR), with the lower and upper bounds of each box corresponding to the 25th and 75th percentiles, respectively
